# Optimized Protocols for the Propagation and Quantification of Infectious Murine Hepatitis Virus (MHV-A59) Using NCTC Clone 1469 and 929 Cells

**DOI:** 10.3390/mps5010005

**Published:** 2022-01-03

**Authors:** Tautvydas Shuipys, Naim Montazeri

**Affiliations:** Department of Food Science and Human Nutrition, University of Florida, Gainesville, FL 32611, USA; tshuipys@ufl.edu

**Keywords:** cell passage, CCL-1, CCL-9.1, coronavirus, COVID-19, MHV, plaque assay, SARS, surrogate

## Abstract

Murine hepatitis virus (MHV) is a non-human pathogen betacoronavirus that is evolutionarily and structurally related to the human pathogenic viruses SARS-CoV, MERS-CoV, and SARS-CoV-2. However, unlike the human SARS and MERS viruses, MHV requires a biosafety level 2 laboratory for propagating and safe handling, making it a potentially suitable surrogate virus. Despite this utility, few papers discussed the propagation and quantification of MHV using cell lines readily available in biorepositories making their implementations not easily reproducible. This article provides protocols for propagating and quantifying MHV-A59 using the recommended NCTC clone 1469 and clone 929 cell lines from American Type Culture Collection (ATCC). More specifically, the methods detail reviving cells, routine cell passaging, preparing freeze stocks, infection of NCTC clone 1469 with MHV and subsequent harvesting, and plaque assay quantification of MHV using NCTC clone 929 cells. Using these protocols, a BSL-2 laboratory equipped for cell culture work would generate at least 6.0 log plaque-forming units (PFU) per mL of MHV lysate and provide an optimized overlay assay using either methylcellulose or agarose as overlays for the titration of infectious virus particles. The protocols described here are intended to be utilized for persistence and inactivation studies of coronaviruses.

## 1. Introduction

Prior to early 2003, human coronaviruses were known to cause only self-limiting, superficial infections resulting in cold symptoms—a stark contrast to the severe diseases caused by coronaviruses in animals [1]. During the past decades, two new human coronaviruses emerged, leading to outbreaks of severe acute respiratory syndrome (SARS) and Middle East respiratory syndrome (MERS). SARS-CoV was the first human coronavirus that caused a severe and fatal disease leading to over 8000 infected individuals and the death of almost 800 people before the outbreak was controlled [2,3,4]. Those infected with MERS-CoV exhibited a higher fatality rate, especially in patients with comorbidities, resulting in 2279 confirmed cases with 806 deaths by 2018 [5,6,7]. Most recently, an outbreak of viral pneumonia in Wuhan, China was identified to be caused by another human coronavirus designated SARS-CoV-2 and has led to the global pandemic known as coronavirus disease 2019 (COVID-19) [8]. As of October 13, 2021, SARS-CoV-2 has infected over 238 million people and resulted in 4.8 million deaths [9]. However, unlike MERS, the presence of comorbidities does not appear to be directly correlated with an increased fatality rate for COVID-19 [10,11].

Given the risk of severe disease and death, the World Health Organization has classified SARS-CoV-2 as a Risk Group 3 agent similar to SARS-CoV and MERS-CoV [12]. Consequently, studies on these viruses must be performed at Biosafety Level 3 (BSL-3) laboratories, which involves strict requirements for containment and special facility design, including further personnel protective equipment, high-efficiency particulate air (HEPA) filtered directional airflow, and laboratories separated from areas with unrestricted access [13]. These requirements limit the number of laboratories that would be qualified to work with these viruses. To circumvent this limitation, research laboratories studying SARS-associated coronaviruses utilize surrogate viruses that can be handled in BSL-1 or BSL-2 laboratories. Typically, surrogates would be used to infer similar characteristics of a target virus while limiting environmental and researcher exposure to potentially hazardous pathogens. Studying the ecology of the target pathogen, its environmental persistence, transmission, and evaluating the efficacy of mitigation strategies are some examples of using the model viruses. Some of the suggested surrogates for highly pathogenic CoV include human coronaviruses such as 229E, NL63, and OC43 as well as animal coronaviruses including feline infectious peritonitis virus, murine hepatitis virus (MHV), and canine coronavirus [14]. While there is currently no consensus on the most optimal coronavirus surrogate, the chosen surrogate should reasonably well represent the target. Based on this, a reasonable starting point is testing the closest evolutionary relative—MHV in the case of SARS-CoV-2.

MHV, similar to the three SARS-associated coronaviruses (SARS-CoV, SARS-CoV-2, and MERS-CoV), belongs to the genus *Betacoronavirus*. MHV, first discovered in 1947, is an enveloped virus with a positive-sense, single-stranded RNA genome [15,16]. Depending on the specific strain, MHV infects the liver and brain of mice allowing for its use in the study of hepatitis and neurological diseases such as demyelination [17,18]. Polytropic strains such as MHV-JHM and MHV-A59 first infect the respiratory system before spreading to other organs, while enterotropic strains such as MHV-DVIM replicate in the intestinal tract [19]. The wide range of MHV strains with different pathologies and transmission routes makes it a versatile virus for study. In the last two decades, the importance of MHV has increased based on its similarity to the SARS-associated coronaviruses. A phylogenic study revealed that SARS-CoV and SARS-CoV-2 belong to the genus *Betacoronavirus* lineage b, MERS-CoV lineage c, and MHV to lineage a [20,21]. This places MHV as a more evolutionarily related virus to the SARS-associated coronaviruses than other potential surrogates such as human coronavirus 229E, feline coronavirus, or transmissible gastroenteritis virus, which are alphacoronaviruses belonging to lineages b, a, and a, respectively. While MHV does bind to a different receptor, CEACAM1, to enter cells compared with ACE2 for SARS-CoV/CoV-2 or dipeptidyl-peptidase 4 (DPP4) for MERS-CoV [22], researchers did find that the S2 subunit of the MHV spike protein shared 40% amino acid identity with SARS-CoV and formed similar architecture [23].

Based on the phylogenic and morphological similarities, some studies have investigated the impact of temperature and humidity on MHV survival [24] as well as the effectiveness of common disinfectants [25] using it as a surrogate for SARS and other coronaviruses. In addition to survival, transmission, and inactivation studies, MHV has even been included in the testing of some potentially antiviral drugs [26,27]. These studies have suggested that MHV responds similarly to the viruses of interest. For this reason, upon the start of the COVID-19 pandemic, MHV was included as one of the recommended surrogates by ASTM International [14]. Despite the utility of MHV, there are very few papers that discuss the protocols for propagating and quantifying the virus or for growing the associated cell lines. Leibowitz, et al. [28] published a collection of detailed protocols, including plaque assay, TCID_50_ assay, propagation, purification, and generation of recombinant viruses. However, the utilized cell lines are not available through commercially accessible repositories such as American Type Culture Collection (ATCC). This creates a key hurdle in research, especially when events such as the COVID-19 pandemic present an urgent need for data. Furthermore, while these cell lines might be obtainable from individual laboratories, issues regarding authentication of the cells and standardization across multiple sources may lead to problems with the reproducibility of published results.

The MHV-A59 (VR-764^TM^), can be obtained directly from ATCC along with NCTC clone 1469 (CCL-9.1^TM^), which is listed as the host cell line. Conversely, there are minimal instructions for propagating the virus, maintaining the host cell line, and quantifying the infectious virus particles. While the NCTC clone 1469 cell line could potentially be used in a tissue culture infectious dose (TCID_50_) assay, the nature of the cell line—a mixture of adherent and suspension cells—and its growth characteristics limit its effective use in a plaque assay. Both TCID_50_ and plaque assays can titrate infectious virus particles; however, plaque assay is a direct, quantitative measurement of the number of plaque-forming units in a virus sample, while TCID_50_ is a qualitative measurement analyzing the ratio of infected and non-infected cells at a specific dilution [29]. Thus, to directly quantify MHV-A59 using a plaque assay, an additional alternative cell line is required. Previous research reported using NCTC clone 929 cells to titer MHV via plaque assay [30,31]; however, details pertaining to the specifics of the assay such as seeding densities, incubation times, or overlays were not adequately provided. Based on this precedent, a plaque assay using NCTC clone 929 (CCL-1^TM^), also obtainable from ATCC, was tested, revealing the cell line optimal for such an application. Overall, the lack of detailed guidelines for working with NCTC clones 1469 and 929 can limit the utility of MHV as a surrogate for SARS-CoV-2. A substantial amount of effort is required for developing protocols to propagate and quantify the virus before any downstream assays may be conducted. The aim of this paper is to remove the barrier, allowing researchers to move forward with experiments by describing detailed protocols for the propagation of the host cell lines NCTC clone 1469 (CCL-9.1^TM^) and NCTC clone 929 (CCL-1^TM^) as well as the propagation and quantification of MHV-A59. The virus particles generated using these protocols can be used in further downstream applications such as environmental persistence studies and evaluation of virus response to mitigation strategies. The proposed protocols represent the authors’ approach under the existing laboratory conditions. Further optimizations may be required when working with other MHV strains and host cell types, alternative reagents, or under different laboratory conditions.

## 2. Experimental Design

**Scheme 1:**
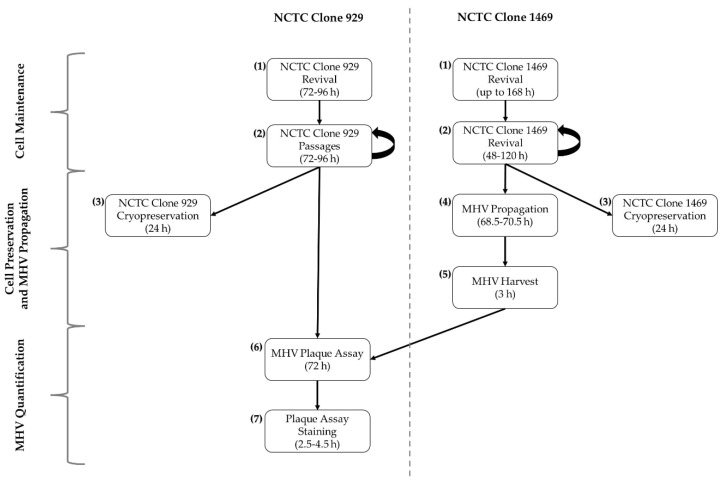
Visual representation of the complete workflow from cell revival to MHV propagation and quantification. The time required for each step is listed in parenthesis. The workflow begins with cell maintenance including (1) revival of frozen cells. Afterwards, (2) the cell lines are passaged until stable cell counts and viability are obtained. At this point, (3) preparation of freeze stocks is recommended before starting (4) MHV propagation, (5) MHV harvesting, and (6) conducting a plaque assay with (7) staining to quantify infectious viral titer.

All virus and cell stocks were procured from American Type Culture Collection (ATCC). The proposed methods used the original frozen stocks with no prior treatments. For the first stage of this protocol, NCTC clone 1469 (CCL-9.1^TM^, *Mus musculus* liver cells) and NCTC clone 929 (CCL-1^TM^, *Mus musculus* areolar adipose cells) were revived from cryopreservation and grown in a 37 °C incubator at 5% CO_2_ for several passages until the cell growth and viability reach stable levels (>90% viability with >0.5–1.0 × 10^6^ live cells/mL). These cell lines are grown in tissue culture flasks, but NCTC clone 1469 requires Dulbecco’s Modified Eagle’s Medium (DMEM) while NCTC clone 929 requires Eagle’s Minimum Essential Medium (EMEM), both to be supplemented with 10% horse serum. NCTC clone 1469 cells serve as hosts for MHV-A59 propagation. While these cells are characterized as adherent by the ATCC, they behave as a mixed population of adherent and suspended cells. This was not impeding for virus propagation but made NCTC clone 1469 cells not ideal for plaque assay. Under our experimental conditions, NCTC clone 1469 cells were not able to form a confluent monolayer sufficient for plaque formation. As a result, NCTC clone 929 cells were utilized for MHV quantification as they proliferate rapidly, produce high cell counts, and form confluent monolayers. However, these cells are also sensitive to high density, with cells that remain at 100% confluency becoming rounded after 2–3 days and sloughing off after 4–7 days. Once both cell lines were established, the NCTC clone 1469 monolayer cells were infected with MHV and monitored over time for cytopathic effects (CPEs). When ca. 90% of cells exhibit CPEs, the propagated virus particles were harvested using a repeated freeze-thaw approach, clarified with centrifugation, and stored at −80 °C until needed. Finally, NCTC clone 929 cells were seeded in 6-well plates at high seeding density to titer the propagated virus particles. Owing to their rapid growth rate, using a lower seeding density for an extended time resulted in inconsistent growth leading to pieces of the monolayer sloughing off prematurely. By using a high seeding density, the cells settle and form a complete monolayer within 24 h. Serial dilutions of the virus are applied to the monolayers, an agarose or methyl cellulose overlay added, and plates incubated for 48 h at 37 °C with 5% CO_2_ to allow plaques to form. Neutral red was used to stain the monolayer when overlaid with agarose or fixed and stained with formaldehyde and crystal violet when overlaid with methyl cellulose, to provide increased contrast for counting of plaques. An overview of this workflow is shown in Scheme 1.

### 2.1. Materials

- 2 mL Internal Threaded Polypropylene Cryogenic Vial (Corning, NY, USA; Cat. No.: 431386)
- 2-Propanol, Certified ACS (Fisher Chemical, Fairlawn, NJ, USA; Cat. No.: A416-1)
- 0.2 mL Polypropylene PCR 8-Tube Strips (USA Scientific, Ocala, FL; Cat. No.: 14024700)
- 15 mL Polypropylene Centrifuge Tubes (Celltreat, Pepperell, MA, USA; Cat. No.: 229412)
- 50 mL Polypropylene Centrifuge Tubes (Celltreat, Pepperell, MA, USA; Cat. No.: 229420)
- Crystal Violet, 1% aqueous solution (Sigma-Aldrich, St. Louis, MO, USA; Cat. No.: V5265)
- Dimethyl sulfoxide, ≥99.5% (GC) (Sigma-Aldrich, St. Louis, MO, USA; Cat. No.: D4540)
- Dulbecco’s Modification of Eagle’s Medium, Powder (Corning, Manassas, VA, USA; Cat. No.: 50003-PB)
- 500 mL Disposable Polyethersulfone (PES) 0.20 μm Filter Units (Fisher Scientific, Rochester, NY, USA; Cat. No.: FB12566504)
- 6-Well Surface-Treated Sterile Tissue Culture Plates (Fisher Scientific, Rochester, NY, USA; Cat. No.: FB012927)
- Formaldehyde, 37% by Weight/Molecular Biology (Fisher BioReagents, Fair Lawn, NJ, USA; Cat. No.: BP531-500)
- Horse Serum, New Zealand origin, not heat-inactivated (Gibco, Penrose, Auckland, New Zealand; Cat. No.: 16050122)
- Methyl Cellulose, viscosity 4000 cP (Sigma-Aldrich, St. Louis, MO, USA; Cat. No.: M0512)
- Minimum Essential Medium Eagle (EMEM), powder (Sigma-Aldrich, St. Louis, MO, USA; Cat. No.: M0643)
- Murine Hepatitis Virus (MHV-A59) (ATCC, Manassas, VA, USA; Cat. No.: VR-764)
- NCTC 135 Medium (Sigma-Aldrich, St. Louis, MO, USA; Cat. No.: N3262)
- NCTC Clone 1469 [derivative of NCTC 721] (ATCC, Manassas, VA, USA; Cat. No.: CCL-9.1)
- NCTC Clone 929 [L cell, L-929, derivative of Strain L] (ATCC, Manassas, VA, USA; Cat. No.: CCL-1)
- Neutral Red, 50% min. dye content (ACROS Organics, NJ, USA; Cat. No.: 229811000)
- 25 cm^2^ Nunc™ EasYFlask™ Cell Culture Flasks (Thermo Scientific, Newington, NH, USA; Cat. No.: 156340)
- 75 cm^2^ Nunc™ EasYFlask™ Cell Culture Flasks (Thermo Scientific, Newington, NH, USA; Cat. No.: 156499)
- Phosphate-Buffered Saline (PBS), pH 7.2 (Gibco, Grand Island, NY, USA; Cat. No.: 20012027)
- Penicillin-Streptomycin-Glutamine (100×), 10,000 U/mL (Gibco, Grand Island, NY, USA; Cat. No.: 10378016)
- SeaPlaque™ Agarose (Lonza, Rockland, ME, USA; Cat. No.: 50100)
- Sodium Bicarbonate, 7.5% solution (Gibco, Grand Island, NY, USA; Cat. No.: 25080-094)
- Sodium Pyruvate Solution, 100 mM (Sigma-Aldrich, St. Louis, MO, USA; Cat. No.: S8636)
- Trypan Blue, 0.4% (*w/v*) in PBS, pH 7.5 (Corning, Manassas, VA, USA; Cat. No.: 25-900-CI)
- Trypsin-EDTA (0.05%), phenol red (Gibco, Grand Island, NY, USA; Cat. No.: 25300054)

### 2.2. Equipment

−80 °C Freezer, Revco™ RLE Series (Thermo Scientific, Newington, NH, USA; Cat. No.: RLE60086A)
- Analytical Balance, Adventurer^®^ Pro (Ohaus, Parsippany, NJ, USA; Cat. No.: AV114)
- Refrigerated Benchtop Centrifuge, Allegra 64R (Beckman Coulter, Indianapolis, IN, USA; Cat. No.: 367586)
- Fixed-Angle Aluminum Rotor, capacity of 6 × 85 mL (Beckman Coulter, Indianapolis, IN, USA; Cat. No.: F0685)
- Biological Safety Cabinet, 1300 Series II, Type A2 (Thermo Scientific, Newington, NH, USA; Cat. No.: 1375)
- Carbon Dioxide (CO_2_), Industrial Grade (Airgas, Radnor, PA, USA; Cat. No.: CD 50)
- CO_2_ Incubator, Heracell™ Vios 160i (Thermo Scientific, Newington, NH, USA; Cat. No.: 50144906)
- TC20™ Automated Cell Counter (Bio-Rad, Hercules, CA, USA; Cat. No.: 1450102)
- Cell Counting Slides for TC20™ Cell Counter (Bio-Rad, Hercules, CA, USA; Cat. No.: 1450011)
- Inverted Phase Contrast Microscope, TMS (Nikon, Melville, NY, USA; Cat. No.: TMS 213971)
- Liquid Nitrogen Cryogenic Storage Vessel (Taylor-Wharton, Baytown, TX, USA; Cat. No.: 34XT)
- LSE™ Compact Centrifuge, capacity of 12 × 15 mL (Corning, NY, USA; Cat. No.: 6756)
- Mr. Frosty™ Freezing Container (Thermo Scientific, Newington, NH, USA; Cat. No.: 5100-0001)
- Precision™ General Purpose Water Bath, capacity of 5 L (Thermo Scientific, Newington, NH, USA; Cat. No.: TSGP05)

## 3. Procedure

### 3.1. Freeze Stock Revival (72–168 h)

1. Temper complete media to 37 °C.
  a. Complete DMEM for NCTC clone 1469 (Section 5.1.1).
  b. Complete EMEM for NCTC clone 929 (Section 5.1.2).
2. Take out the frozen vial of cells from the liquid nitrogen storage vessel and immediately transfer to a 37 °C water bath.
  a.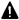 CRITICAL STEP—Upon receiving cells from ATCC, they should be immediately stored in the vapor phase of the liquid nitrogen cryogenic storage vessel (−135 °C to −190 °C).
  b. Avoid submerging the vial in the water bath to prevent any water from entering inside the vial.
3. Let the vial almost completely thaw (2–3 min).
4. In a biosafety cabinet, transfer the vial content into a 15 mL centrifuge tube.
5. Add 5 mL pre-warmed media dropwise and pipette up and down gently to break clumps.
6. In a 0.2 mL tube, combine 10 µL of cell suspension with 10 µL of trypan blue and pipette gently to mix. Load the cell counting slide with 10 µL of the mixture then insert into the TC20™ cell counter to enumerate the Total and Live cells.
  a. Ensure the slide is clean without any dust or fingerprints.
  b.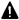 CRITICAL STEP—Ensure no air bubbles are trapped in the cell counting slide to obtain an accurate reading.
7. Seed a 25 cm^2^ flask with:
  a. For NCTC clone 1469: 1.0 × 10^6^ of live cells (the whole suspension may be required).
    i. Incubate cells for up to 7 d in a 37 °C incubator, 5% CO_2_. Since NCTC clone 1469 is composed of a mixed population of cells, there will be a variety of weakly adherent and suspended round cells as well as adherent epithelial-like cells. Cells can be passaged after 3–4 d if the confluency (round, weakly adherent cells plus epithelial-like cells) is >60%. Otherwise, the cell culture medium should be replaced after 3–4 d and the flask incubated until 7 d post revival.
      1. To replace the medium:
        a. Collect the old cell culture medium in a 15 mL centrifuge tube.
        b. Add 1 mL of fresh medium (tempered to 37 °C) to the flask to prevent any adhered cells from drying out.
        c. Centrifuge the old cell culture medium at 300× *g* (RT) for 5 min to collect the suspended cells.
        d. Gently resuspend the pellet in 4 mL of fresh medium and add back to the flask.
  b. For NCTC clone 929: 5.0 × 10^5^ of live cells (the whole suspension may be required).
    i. Incubate cells for up to 3–4 d in a 37 °C incubator, 5% CO_2_ until 80–95% confluent.
8. The total volume of cell suspension and cell culture medium should be ca. 5 mL.

### 3.2. Cell Passage

#### 3.2.1. NCTC Clone 1469 (48–120 h)

**Figure 1:**
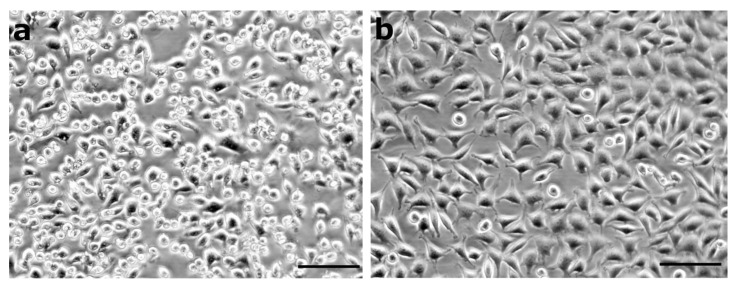
Typical appearance and confluency of cells prior to cell passage. (**a**) NCTC clone 1469; cell culture medium was removed prior to this photo (see Section 3.2.1 step 22). (**b**) NCTC clone 929. Scale bar represents 100 µm.

1. Temper PBS, trypsin, and complete DMEM medium to 37 °C in a water bath.
2. For each passage, prepare at least two flasks (one flask as a backup).
3. Once the cells reach appropriate confluency (Figure 1a), remove the culture medium by decanting or pipetting and transfer to a 50 mL centrifuge tube.
4. While holding the flask at a 45° angle, wash the cell monolayer by gently dispensing PBS (tempered to 37 °C) using a serological pipette to remove any residual serum that will interfere with trypsinization.
  a. Recommended volumes: 5 mL PBS for a 25 cm^2^ flask and 10 mL PBS for a 75 cm^2^ flask
  b.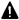 CRITICAL STEP—Only wash once and avoid shaking to prevent dislodging too many of the weakly adherent cells. Some NCTC clone 1469 cells will be dislodged, as evidenced by the wash PBS becoming slightly cloudy, but efforts should be made to prevent too many of the cells from being dislodged.
5. Rotate the flask so that the wash PBS accumulates in the corner opposite the cell monolayer (typically one of the top corners) and remove using the same serological pipette.
  a. Discard the PBS wash into the waste container.
    i. Bleach the waste container before disposal.
  b.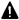 CRITICAL STEP—Avoid touching the bottom of the flask with the pipette and accidentally scraping off cells.
6. Add trypsin (tempered to 37 °C) to the flask using a serological pipette.
  a. 2 mL for a 25 cm^2^ flask.
  b. 3 mL for a 75 cm^2^ flask.
7. Incubate the flask at room temperature until most cells are rounded/dislodged by visualizing under a microscope (3–5 min).
  a. May require gentle to moderate rocking or gentle tapping of the flask to dislodge the cells.
8. Neutralize trypsin using the culture medium collected in step 3.
  a. 5 mL for a 25 cm^2^ flask.
  b. 9 mL for a 75 cm^2^ flask.
9. Using a 10 mL pipette, wash the cells from the flask by holding the flask at a 45° angle and pipetting (5–10 times) gently down the bottom of the flask.
  a.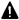 CRITICAL STEP—Avoid excessive or rigorous pipetting, which may result in cell damage.
  b. The bottom of the flask should look clear against a light when all the cells have been washed off. Presence of a translucent layer/patches indicates the presence of cells that have not been washed off.
10. Transfer the suspension to a 50 mL centrifuge tube combining with the cell suspension collected in step 3.
11. Pellet down the cells by centrifuging for 5 min at 280× *g*, RT.
  a. A cell pellet should be visible.
12. Carefully discard the supernatant by pipetting or decanting.
13. Resuspend the pellet in 9 mL fresh complete medium and pipetting up and down to break down the large clumps.
14. In a 0.2 mL tube, count total and live cells, as explained in Section 3.1 step 6.
15. Calculate the volume of cell suspension required for a seeding density of 7.9 × 10^6^ live cells.
16. Fill the 75 cm^2^ flask in an upright position with a total of 20 mL of culture medium less the amount of cell suspension calculated in step 15.
17. Add the volume of cell culture calculated in step 15 to the flask.
18. Tighten the cap if vented. Leave it slightly loose if not vented.
19. Rock the flasks in four directions (north-south, east-west) a few times before placing into the incubator.
  a.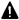 CRITICAL STEP—Avoid swirling the flasks as that will result in the cells concentrating around the perimeter of the flask leading to uneven growth.
20. Incubate at 37 °C, 5% CO_2_ for 2 d.
21. After 2 d, repeat steps 1–2.
22. When removing the cell culture medium, discard instead of retaining.
  a. Note: we found that alternating keeping vs. discarding suspension cells in the old cell culture medium resulted in the best compromise between the number of adherent cells and total cell counts.
  b. When propagating MHV, the old cell culture medium is removed prior to the addition of the virus suspension. As a result, by discarding the old cell culture medium at this stage, the cell counts from this passage could be used to estimate MOI calculations.
23. Repeat steps 4–14.
24. Calculate a cell seeding density of 4.0 × 10^6^ cells for a 75 cm^2^ flask.
25. Repeat steps 16–19.
26. Incubate at 37 °C, 5% CO_2_ for 5 d.

#### 3.2.2. NCTC Clone 929 (72–96 h)

1. Temper PBS, trypsin, and complete EMEM to 37 °C in a water bath.
2. For each passage, prepare at least two flasks (one flask as a backup).
3. Once the cells reach 80–95% confluency (see Figure 1b), remove the culture medium by decanting or pipetting and discarding it in a waste container.
4. While holding the flask at a 45° angle, wash the cell monolayer by gently dispensing PBS (tempered to 37 °C) using a serological pipette to remove any residual serum that may interfere with trypsinization.
  a. Use 5 mL PBS for a 25 cm^2^ flask and 10 mL PBS for a 75 cm^2^ flask.
  b. Only wash once and avoid shaking to prevent dislodging cells.
5. Rotate the flask so that the wash PBS accumulates in the corner opposite the cell monolayer (normally one of the top corners) and remove using the same serological pipette.
  a. Discard the PBS wash into the waste container.
    i. Bleach the waste container before disposal.
  b.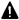 CRITICAL STEP—Avoid touching the bottom of the flask with the pipette and accidentally scraping off cells.
6. Add trypsin (tempered to 37 °C) to the flask using a serological pipette.
  a. 2 mL for 25 cm^2^ flasks
  b. 3 mL for 75 cm^2^ flasks
7. Incubate the flask at room temperature until most cells are rounded by visualizing under a microscope (2–4 min).
8. Neutralize trypsin using fresh complete EMEM (tempered to 37 °C).
  a. 5 mL for a 25 cm^2^ flask
  b. 9 mL for a 75 cm^2^ flask
9. Using a 10 mL pipette, wash the cells from the flask by holding the flask at a 45° angle and pipetting (5–10 times) gently down the bottom of the flask.
  a.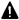 CRITICAL STEP—Avoid excessive or rigorous pipetting, which may result in cell damage.
  b. The bottom of the flask should look clear against a light when all the cells have been washed off. Presence of a translucent layer/patches indicates the presence of cells that have not been washed off.
10. Transfer the suspension a 15 mL centrifuge tube.
11. Pellet down the cells by centrifuging for 5 min at 280× *g*, RT.
  a. A cell pellet should be clearly visible.
12. Carefully discard the supernatant by pipetting or decanting.
13. Resuspend the cells in 9 mL medium by pipetting up and down to break up the pellet and large clumps.
14. In a 0.2 mL tube, combine 10 µL of cell suspension with 10 µL trypan blue and mix gently by pipetting. Pipet 10 µL of the mixture into a cell counting slide then count the total and live cells.
  a. Ensure the cell counting slide is clean without any dust or fingerprints.
  b.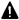 CRITICAL STEP—Ensure no air bubbles are trapped in the cell counting slide to obtain an accurate reading.
15. Calculate cell seeding densities for a 75 cm^2^ flask:
  a. For a passage after 72 h: 1.5 × 10^6^ cells.
  b. For a passage after 96 h: 1.0 × 10^6^ cells.
  c. Note: as the passage number increases, the cells will become more acclimated to laboratory conditions resulting in faster growth. As a result, the seeding density may need to be decreased to ensure the monolayer does not become too dense. NCTC clone 929 is sensitive to high density with the cells first turning rounded then sloughing off. Initially, the round cells are still alive, but viability will decrease soon after.
  d. It is recommended to always run a backup flask with 1:10 seeding density.
16. Fill the 75 cm^2^ flask in an upright position for a total of 20 mL of medium less the amount of cell suspension calculated in step 15.
17. Add the volume of cell culture calculated in step 15 to the flask.
18. Tighten the cap if vented. Leave it slightly loose if not vented.
19. Rock the flasks in four directions (north-south, east-west) a few times before placing into the incubator.
  a.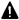 CRITICAL STEP—Avoid swirling the flasks as this will result in the cells concentrating around the perimeter of the flask leading to uneven growth.
20. Incubate at 37 °C, 5% CO_2_ for 3–4 d or until confluency reaches 80–95%.

### 3.3. Cryopreservation (24 h)

1. Grow cells until at least 0.5 × 10^6^ cells/mL with >90% viability is reached (passage 4 to 7).
2. Proceed with cell passage steps 3–12 as described in Section 3.2.1 for NCTC clone 1469 or Section 3.2.2 for NCTC clone 929.
3. Resuspend the cells using 9 mL of cold (ca. 4 °C) freezing medium and pipette up and down to break clumps.
  a. DMEM freezing medium for NCTC clone 1469
  b. EMEM freezing medium for NCTC clone 929
4. Perform cell count as explained above.
5. Adjust the cell density to ca. 1.0 × 10^6^ live cells/mL.
6. Using a 10 mL serological pipette, add 1 mL cell suspension to each 2 mL cryogenic vial.
  a. Ensure to screw caps tight.
7. Place tubes into Nalgene Mr. Frosty freezing container after preparing container using manufacturer’s instructions. Then place Mr. frosty container into the −80 °C freezer for 24 h.
8. After 24 h, transfer cryogenic vials to nitrogen vapor phase for long-term storage.
9. After a few days, revive one of the frozen cell vials to ensure cell viability.

### 3.4. MHV Propagation

#### 3.4.1. Infection of Cells (68.5–70.5 h)

1. Passage NCTC clone 1469 cells into a 75 cm^2^ flask at a seeding density of 7.0 × 10^6^ live cells using complete DMEM.
  a. 48 h later, the flask should be 60–80% confluent.
2. Temper base and complete NCTC-135 medium to 37 °C.
3. Thaw a vial of MHV-A59 (ATCC VR-764) in a 37 °C water bath (it takes ca. 3 min).
4. Prepare dilutions of the virus in a 15 mL centrifuge tube using base NCTC-135 medium (final volume 2.5 mL for a 75 cm^2^ flask) for the desired MOI.
  a. We found that an MOI between 7 × 10^−4^ and 3 × 10^−5^ produced an optimum viral titer.
    i. See Table 1 for example MOI’s and titers of virus produced.
  b. When using the original frozen stock, the ATCC recommends [32]:
    i. For titer between 3–6 log TCID_50_, dilute virus 1:100 before inoculation.
    ii. For titer greater than 6 log TCID_50_, dilute virus 1:1000 before inoculation.
5. Remove cell culture medium from flask and add 2.5 mL of the diluted virus inoculum.
  a. Gently rock forward and backward to distribute evenly.
6. Incubate flask at 37 °C, 5% CO_2_ for 1 h.
  a. Gently rock flask forward and backward every 15 min.
  b. Ideally, this would be in an incubator separate from the one used for normal cell culture to prevent accidental viral contamination.
7. After 1 h, remove the virus inoculum and add 20 mL of complete NCTC-135 medium (5% Horse Serum).
8. Place the flask back in the incubator (37 °C, 5% CO_2_) for 18–20 h until the cytopathic effects (CPE) become visible.
9. CPE for this virus is the formation of syncytium: fusion of cells into large, multinucleate cells. These will be detached from the flask and floating in suspension.

#### 3.4.2. Harvesting MHV (3 h)

**Figure 2:**
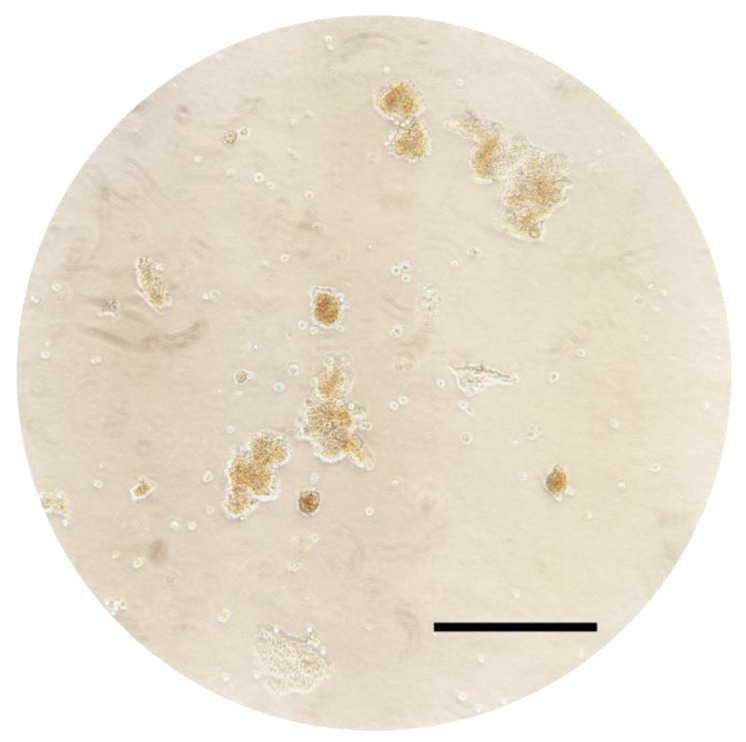
Cytopathic effect (CPE) of MHV infected NCTC clone 1469 cells after 19 h. Infected cells form syncytium: a multinucleated mass formed by the fusion of cells. Scale bar represents 1 mm.

1.
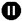
AUSE STEP—When 90% of cells exhibit cytopathic effects (see Figure 2), roughly 18–20 h post-infection, move the flask to the −80 °C freezer.
  a. The frozen flask can be stored until a later date before proceeding to the rest of the steps.

2. After the flask has frozen (at least 30 min), move the flask immediately into a large water bath at 37 °C.
  a.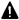 CRITICAL STEP—Avoid submerging the cap.
3. Allow to thaw for ca. 10 min, then remove and vigorously shake the flask for ca. 30 s to help lyse the cells.
4.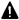 CRITICAL STEP—De-gas the flask in the biosafety cabinet by briefly opening the cap to prevent any occasional pressure buildup during the freeze-thaw.
5. Place the flask back into the −80 °C freezer for at least 30 min.
6. Repeat steps 2–5 two more times.
7. Following the third freeze-thaw cycle, collect as much as the cell culture lysate as possible using a serological pipette moving it into a 50 mL centrifuge tube.
8. Centrifuge the tube at 12,000× *g* for 20 min at 18 °C.
9. Carefully decant the supernatant into a new centrifuge tube.
  a. OPTIONAL STEP—After centrifuging, pass virus supernatant through a 0.2 µm filter to further remove the debris.
10. Quick freeze the vials by placing them into the liquid nitrogen storage vessel for ca. 30 min before moving to the −80 °C freezer for long-term storage.

### 3.5. MHV Plaque Assay

#### 3.5.1. NCTC Clone 929 Cell Preparation (24 h)

1. Follow the NCTC clone 929 passage protocol (3.2.2) to harvest the cells from the flask (steps 1–14).
2. Add 9.5 × 10^5^ NCTC clone 929 cells per well into a 6-well plate with a total volume of 2.5 mL.
3. Rock the plates in four directions (north-south, east-west) to ensure the distribution of the cells.
  a.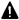 CRITICAL STEP—Avoid swirling the plates as this will result in the cells concentrating around the perimeter of the well leading to uneven growth.
4. Incubate the plates for 24 h at 37 °C, 5% CO_2_ at which point there should reach >90% confluency.
  a. Note: there are two methods that can be used at this point to set up an MHV plaque assay. In Section 3.5.2, a solid agarose overlay is applied after MHV infection of the cells followed by Section 3.5.3 to stain the cells using Neutral Red dye. Alternatively, Section 3.5.4 employs a methyl cellulose overlay followed by Section 3.5.5 to fix and stain the cells using formaldehyde with crystal violet. See 4. Expected results for discussion of the two approaches.

#### 3.5.2. NCTC Clone 929 Infection with MHV Using a Solid Overlay (48 h)

1. Once the cells are >90% confluent, thaw an MHV virus aliquot in a water bath at 37 °C (ca. 2–3 min).
2. Prepare serial dilutions of the virus in base NCTC-135 medium.
3. Move the 6-well plates to the infectious biosafety hood and aspirate the cell culture medium into a waste container.
  a.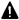 CRITICAL STEP—Work with up to three 6-well plates at a time to prevent the cell monolayer from drying out.
4. Gently add 500 µL of the virus suspension to each well. Ensure the pipette tip touches the inside wall of each well.
5. Rock the plate in four directions (north-south, east-west) to ensure the virus suspension covers the monolayer.
6. Incubate the plates for an hour in the incubator (37 °C, 5% CO_2_), rocking the cells gently every 15 min to distribute the virus evenly.
7. While the plates are incubating, prepare the overlay by melting 3% SeaPlaque™ agarose then tempering to 39–42 °C along with 2 × Plaque Assay Medium in a water bath.
  a. Once both are tempered, combine in a 1:1 ratio depending on the number of plaque assay plates. You will need 2.5 mL per well or 15 mL per 6-well plate.
  b.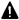 CRITICAL STEP—Ensure that the melted agarose has cooled to 39–42 °C before combining with the plaque assay medium.
8. Gently add 2.5 mL of the above overlay to each well.
9. Leave plates in the biosafety cabinet for ca. 15 min until the agarose solidifies.
10. Place the plates in the incubator (37 °C, 5% CO_2_) for 48 h until the plaques are visible.

#### 3.5.3. Staining plaque assay plates with solid overlay (2.5–4.5 h)

**Figure 3:**
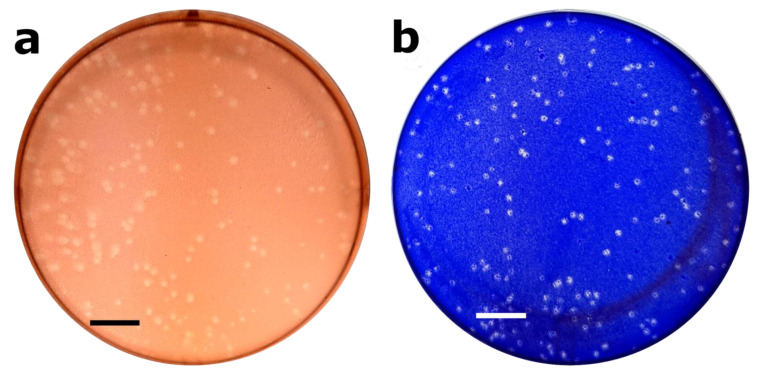
Example of MHV plaque assay wells in titration of two separate virus samples. (**a**) Solid agarose overlay; 4 h after staining with Neutral Red; infected with MHV (5.49 log PFU/mL) at a 10^−3^ dilution. (**b**) Methyl cellulose overlay; 4 h after fixing/staining with crystal violet in formaldehyde; infected with MHV (6.59 log PFU/mL) at a 10^−4^ dilution. The different dilutions of MHV shown in (**a**,**b**) were to present a similar countable range of plaques across the two virus samples. Scale bar represents 5 mm.

1. Once plaques are faintly visible (roughly 48 h after infection), prepare 0.04% *v/v* Neutral Red in PBS (pH 7.2).
2. Stain the wells by adding 1 mL of 0.04% Neutral Red to each.
3. Place the plates back into the incubator (37 °C, 5% CO_2_) for 2–4 h.
4. Remove the neutral red stain.
5. Flip over the plate and count the plaques per well (see Figure 3a).
6. Virus titer is calculated using the following Equation (1):
  (1) $$\mathrm{Virus}\;\mathrm{titer}\;(\mathrm{PFU}/\mathrm{mL})=\frac{\mathrm{Number}\;\mathrm{of}\;\mathrm{Plaques}}{\mathrm{Dilution}\;\times \;\mathrm{Volume}\;\mathrm{of}\;\mathrm{Virus}\;\mathrm{Added}\;\left(\mathrm{mL}\right)}$$

#### 3.5.4. ALTERNATIVE: NCTC Clone 929 Infection with MHV Using a Methyl Cellulose Overlay Solution (48 h)

1. Prepare 0.8% Methyl Cellulose overlay medium by combining a 1.6% *w/v* methyl cellulose solution and 2× Plaque Assay medium in a 1:1 ratio and temper to 37 °C in a water bath (see Section 5.2.5 for details).
2. Follow steps 1–6 from Section 3.5.2.
3. Gently add 2.5 mL of the methyl cellulose overlay medium.
4. Place the plates in the incubator (37 °C, 5% CO_2_) for 48 h until the plaques are visible.
  a. Avoid moving the plates during the incubation period and disrupting the fluid overlay.

#### 3.5.5. ALTERNATIVE: Fixing and Staining Plaque Assay Plates Overlayed with Methyl Cellulose (2.5–4.5 h)

1. Once plaques are faintly visible (roughly 48 h after infection), prepare fresh fixing/staining solution (see Section 5.2.6 details).
  a.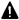 CRITICAL STEP—Keep this solution in the dark until ready for use.
2. In a fume hood, remove the methyl cellulose overlay using a serological pipette and add 1 mL of the above formaldehyde-crystal violet fixing/staining solution.
3. Leave the plates in the fume hood for 2–4 h.
4. Aspirate the fixing/staining solution.
5. Gently rinse the wells using tap water until the rinsate runs clear.
6. Flip over the plate and count the plaques per well (see Figure 3b).
7. Calculate titer using Equation (1) from Section 3.5.3.

## 4. Expected Results

When established, both NCTC clone 1469 and clone 929 exhibit consistent cell counts and viability. For NCTC clone 1469, total live cells and percent viable cells averaged 2.88 × 10^7^ and 95.8% when suspension cells were retained and 8.87 × 10^6^ and 99.4% when suspension cells were discarded in passaging 75 cm^2^ flasks. For NCTC clone 929, an average of 1.56 × 10^7^ total live cells with a 99.9% viability were obtained when passaging 75 cm^2^ flasks. While NCTC clone 1469 cell culture medium tended to discolor to orange/yellow (potentially indicating acidification), we did not observe any negative impact on the cell counts or viability. On the contrary, this cell line is relatively forgiving and can be kept for 7 days between passages without any detrimental effects. Discoloration may be avoided by adjusting sodium bicarbonate concentration, but the cells’ growth rate may be impacted. In such cases, further optimization such as changing seeding density, incubation time, and serum concentration may be required to obtain maximal cell growth and attachment.

The turnaround time was quick for MHV propagation, taking 18–20 h for 90% of cells in a 75 cm^2^ flask to be infected. The clear cytopathic effect also makes it simple to monitor cell infection over time. A titer of about 6 log PFU/mL can be harvested from a 75 cm^2^ containing ~20 mL lysate (see Table 1 for example titers and MOIs). Titer can be further improved through MOI adjustment according to the cells’ growth rate and by incorporating additional purification and concentration steps (not discussed here). We also present two options for setting up a plaque assay: using a solid overlay and neutral red staining or a fluid methyl cellulose overlay and fixing/staining with crystal violet-formaldehyde. While the methyl cellulose method resulted in a stark contrast between the clear plaques and deep blue monolayer, we find that it is easier to count plaques using the solid overlay-neutral red method. However, we found both protocols equally effective in quantifying infectious MHV titer. The other benefit of the solid overlay-neutral red method is no generation of formaldehyde waste that needs to be disposed of according to chemical hazardous waste regulations. On the other hand, the downside to this method is needing to prepare the agarose ahead of time, melting then cooling prior to combination with plaque assay medium, and waiting for the overlay to solidify before the plates are placed back in the incubator. Regardless of which method is used, the turnaround time from seeding 6-well plates to counting plaques is three days allowing relatively rapid generation of results.

With the COVID-19 pandemic showing no signs of abating, there is an ever-present need for more details about SARS-CoV-2’s characteristics, persistence on various environmental surfaces, resistance to conventional and novel disinfection methods, and risk of transferring virus particles from contaminated fomites to other clean surfaces. Such research can be conducted directly using SARS-CoV-2 but carries additional risk for the researchers and requires specialized facilities that few may have access to. MHV, as the closest evolutionary relative to SARS-CoV-2, stands poised as a potential BSL-2 surrogate allowing such research to proceed in the absence of BSL-3 capacity and risk. The protocols presented in this article aimed to fill a gap of knowledge regarding the propagation and quantification of MHV using cell lines obtainable from cell repositories. Thereby, reducing the amount of time and optimization required by labs before proceeding to downstream experiments.

## 5. Reagents Setup

### 5.1. Cell Culture Media

#### 5.1.1. DMEM Complete Medium for NCTC Clone 1469, 500 mL

- 6.74 g DMEM powder (Corning #50-003-PB).
- 10 mL 7.5% Sodium Bicarbonate (Gibco #25080094)—for a final concentration of 1.5 g/L.
- 490 mL Cell Culture Grade Water (or autoclaved deionized water).
- Filter-sterilize the above base medium using a 0.2 µm vacuum filter, replace 55 mL of the medium with:
  ○ 5.0 mL 100× Penicillin-Streptomycin (Gibco #15140-122).
  ○ 50.0 mL Horse Serum (Gibco #16050-122)—for a final concentration of 10%.

#### 5.1.2. Complete EMEM for NCTC Clone 929, 500 mL

- 4.8 g EMEM powder (Sigma #M0643).
- 14.7 mL 7.5% Sodium Bicarbonate (Gibco #25080094).
- 5.0 mL 100 mM Sodium Pyruvate (Sigma #S8636).
- 480.3 mL Cell Culture Grade Water (or autoclaved deionized water).
- Filter-sterilize the above base medium using a 0.2 µm PES vacuum filter, replace 55 mL of the medium with:
  ○ 5.0 mL 100× Penicillin-Streptomycin (Gibco #15140-122).
  ○ 50.0 mL Horse Serum (Gibco #16050-122) for a final concentration of 10%.

#### 5.1.3. Cell Freezing Medium, 50 mL

- To 42.275 mL of base medium removed above (DMEM for NCTC clone 1469, EMEM for NCTC clone 929), add 2.5 mL of DMSO (Sigma #D4540-100ML). Filter-sterilize using a 0.2 µm vacuum filter, then add the following to make complete:
  ○ 0.475 mL 100× Penicillin-Streptomycin (Gibco #15140-122).
  ○ 4.75 mL Horse Serum (Gibco #16050-122).

#### 5.1.4. NCTC-135 Medium for MHV Propagation, 500 mL

- 4.7 g NCTC-135 powder (Sigma # N3262).
- 14.7 mL 7.5% Sodium Bicarbonate—for a final concentration of 2.2 g/L (Gibco #25080094).
- 485.3 mL Cell Culture Grade Water (or autoclaved deionized water).
- Filter-sterilize the above base medium using a 0.2 µm PES vacuum filter. To prepare NCTC-135 medium with 5% Horse Serum (100 mL), combine 95 mL of the filter-sterilized NCTC-135 base medium with 5 mL Horse Serum (Gibco #16050-122).

### 5.2. Plaque Assay Media and Reagents

#### 5.2.1. 2× EMEM for Plaque Assay, 500 mL

- 9.6 g EMEM powder (Sigma #M0643).
- 29.4 mL 7.5% Sodium Bicarbonate (Gibco #25080094).
- 10.0 mL 100 mM Sodium Pyruvate (Sigma #S8636).
- 460.6 mL Cell Culture Grade Water (or autoclaved deionized water).
- Filter-sterilize the above base medium using a 0.2 µm PES vacuum filter, replace 30 mL of the medium with:
  ○ 10.0 mL 100× Penicillin-Streptomycin (Gibco #15140-122).
  ○ 20.0 mL Horse Serum—for a final concentration of 4% (Gibco #16050-122).

#### 5.2.2. 3% SeaPlaque Agarose, 500 mL

- 15 g SeaPlaque Agarose (Lonza #50100).
- 500 mL deionized water.
- Autoclave to sterilize and store at room temperature. Prior to use in a plaque assay, melt using a microwave or in hot water. Can also aliquot the autoclaved agarose into 5 sterile 100 mL bottles.

#### 5.2.3. 1% Neutral Red, 100 mL

- 1 g Neutral Red (ACROS #229811000).
- 100 mL PBS, pH 7.2 (Gibco #20012-027).
- Dissolve then filter-sterilize using a 0.2 µm vacuum filter. Store in the dark.

#### 5.2.4. 1.6% Methyl Cellulose, 250 mL

- 4 g Methyl cellulose, viscosity 4000 cP (Sigma M0512).
- 250 mL DI water.
- Autoclave the methyl cellulose powder in a 500 mL bottle with a stir bar. Autoclave the water separately in another bottle. When the water has cooled enough to handle safely (roughly 50–60 °C), add 250 mL of the water to the methyl cellulose. Place on a stir plate at room temperature for 5 min, then move the bottle and stir plate into a refrigerator or incubator set to 4–6 °C. Stir 2–4 h or until the solution is clear; store refrigerated.

#### 5.2.5. 0.8% Methyl Cellulose Overlay Medium, 500 mL

- To the 250 mL of 1.6% Methyl Cellulose from Section 5.2.4, add 250 mL of 2× medium for plaque assay from Section 5.2.1 in a biosafety cabinet. Stir to mix well.

#### 5.2.6. Fixing/Staining Solution, 100 mL

- 10 mL Formaldehyde, 37% by Weight (Fisher BioReagents #BP531-500).
- 10 mL Crystal Violet, 1% aqueous solution (Sigma-Aldrich #V5265).
- 80 mL DI water.
- Prepare in a fume hood and store in the dark until use.

## Figures and Tables

**Table 1:** Tested MOIs and resulting MHV titer after harvest across two trials.

MOI	Avg. Titer (Log PFU/mL)
3 × 10^−2^	5.67
3 × 10^−3^	6.20
7 × 10^−4^	6.43
3 × 10^−5^	6.43

## Data Availability

The data presented in this study are available on request from the corresponding author.
